# Case report: persistent double dorsal aorta

**DOI:** 10.1590/1677-5449.202301502

**Published:** 2024-05-10

**Authors:** Paulo Henrique Alves Togni, Ernani Alves de Oliveira, Eduardo Milani Mora, Paulo Eduardo Borher Moreira, Bruno Previdelli Coghi, Guilherme Augusto Paro

**Affiliations:** 1 Centro Universitário Padre Albino – UNIFIPA, Catanduva, SP, Brasil.

**Keywords:** anatomic variant, abdominal aorta, computed tomography, congenital anomaly

## Abstract

Persistent double dorsal aorta is an extremely rare congenital anomaly, with only 13 cases published to date. The objective of this study is to present this embryological variant as observed in the abdominal aorta of a patient. The anatomical description was written up on the basis of a review of electronic medical records and imaging exams. The patient in this case was an elderly 79-year-old man who presented at emergency with pain at rest in the left lower limb. He was admitted and laboratory tests and imaging exams were ordered. The variation was an imaging finding observed on angiotomography, consisting of complete separation of the abdominal aorta into two portions - a ventral and a dorsal, with different calibers – at the level of the third lumbar vertebra. There was also an anomalous origin of the inferior mesenteric artery.

## INTRODUCTION

Congenital aberrations and variants of the aortic arch constitute a wide spectrum of developmental anomalies, are relatively common, occurring in 0.5 to 3% of the population, and may or may not be associated with heart defects.^1,2^ Notwithstanding, embryological defects of the dorsal aorta are exceptional.^3^

Persistent double dorsal aorta is an extremely rare anomaly. The first case was described in 1975 by Mosquera and Micarelli^4^ and since then only a few more cases have been reported. Two variants are described in the literature. The pattern most often observed consists of complete separation of two portions of the dorsal aorta. In the other variant, the descending aorta has a double lumen because of the presence of central dividing septum.^3^

This project was approved by the Research Ethics Committee at the Centro Universitário Padre Albino (UNIFIPA), under Ethics Appraisal Submission Certificate number 68416423.0.0000.5430 and consolidated opinion number 5.994.485.

## CASE DESCRIPTION

The patient was a white, 79-year-old male. He was admitted via emergency because of pain at rest in the left lower limb, which had worsened approximately 10 days previously. Physical examination found the patient in good general health, with good color and hydration and peripheral perfusion preserved. There was necrosis of the fifth toe of the left foot, with no signs of inflammation. The patient had a personal history of systemic arterial hypertension and peripheral arterial occlusive disease and was a long-term smoker. Initially, laboratory tests and imaging exams were ordered and the patient was admitted. Once admitted, a 60 mg dose of enoxaparin was administered by injection with a 0.6 mL syringe, in addition to analgesia with a 100 mg ampoule of Tramadol and a 1g ampoule of Dipyrone, both injectable, and the left lower limb was warmed. Laboratory tests showed sodium at 136 mmol/L, with no other abnormalities. Doppler ultrasound of the left-side arteries revealed monophasic flow in the common femoral artery, with occlusion of a proximal and medial segment of the femoral artery. The popliteal artery had monophasic flow; the tibial and fibular arteries had monophasic flow, with occlusion of a distal segment of the posterior tibial. The patient remained in hospital for 13 days in a standard ward for clinical treatment and general care. While in hospital, physiotherapy was administered for the peripheral vascular dysfunctions and for the respiratory disorder without systemic complications. On the seventh day in hospital, aortography and arteriography of the left lower limb were ordered, showing the abdominal aorta with normal morphology and flow and diffuse parietal irregularities.

**Figure 1:**
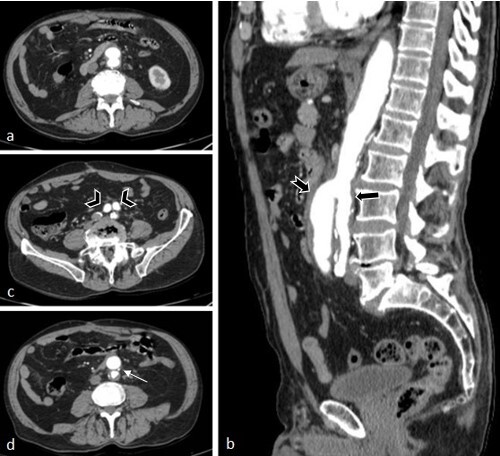
(a) Axial computed tomography (TC) images with an abdominal window; (b) Sagittal CT image with an abdominal window showing inferior duplication of the abdominal aorta with an anterior unit (tailed arrow) and a posterior unit (simple arrow), from the level of L3; (c) Axial CT image with an abdominal window showing inferior duplication of the abdominal aorta, with an anterior unit with a caliber of 2.6 cm, bifurcating into the external iliac arteries (arrowhead); and a posterior unit with a caliber of 2.2 cm, bifurcating into internal iliac arteries (thrombosed in this study); (d) Axial CT image with an abdominal window showing duplication of the inferior portion of the abdominal aorta, with the posterior unit giving rise to the inferior mesenteric artery (thin arrow).

**Figure 2:**
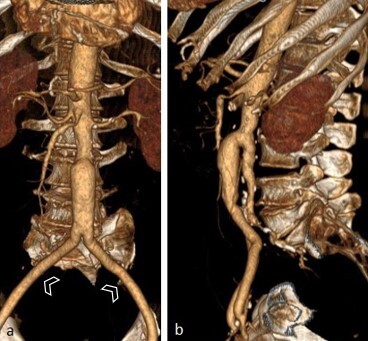
Serial volume-rendered CT images in a frontal view (a) and left lateral view (b), showing duplication of the inferior portion of the abdominal aorta, which bifurcates into the external iliac arteries (arrowheads) and the posterior portion, bifurcating into the internal iliac arteries (thrombosed in this study).

On the 13th day, the patient’s left lower limb had not improved, despite the clinical treatment. The necrosis persisted and there was now discrete yellow secretion, with no hyperemia or local heat, and pulses were not palpable. In order to assess the possibility of transfer to a specialist service in a nearby town, computed angiotomography was ordered on the 13th day. Axial angiotomography slices were acquired of the abdomen after intravenous injection of contrast medium, on which the only remarkable findings were the arterial phase showing opacification of the abdominal aorta and duplication of its inferior portion. The anterior portion had a caliber of 2.6 cm and bifurcated into the external iliac arteries. The posterior portion had a caliber of 2.2 cm, giving rise to the inferior mesenteric artery, which was partially thrombosed and bifurcated into the internal iliac arteries (Figures 1 and 2). The internal iliac arteries were partially thrombosed, more notably on the right, and their distal branches were partially perfused by collaterals from the ipsilateral external iliac arteries. The posterior intercostal arteries originated directly from the thoracic aorta segment. No anomalies of other sites were observed on tomography. A decision was taken to transfer the patient to the specialist service the same day and he was prescribed fasting from 6pm onwards. He was followed-up by a care team in the nearby town.

## DISCUSSION

Persistence of a double dorsal aorta is an extremely rare condition, with just 12 cases described to date.^3,5^ A bibliographic review conducted by Mills and Gest^3^ compiled 11 cases from 1975 to 2016 and just one other case was reported in 2022, by Hurtado et al.^5^

In the embryology of formation of the cardiovascular system, the primary embryonic vessels are the primitive right and left aortas, which connect cranially to the nascent heart via outlet tracts. The caudal extremities elongate via connection to the vascular plexus in the splenic mesoderm. These two paired dorsal aortas are below the lateral plate mesoderm and run the entire length of the embryo. Soon after they appear as separate paired vessels, the dorsal aortas undergo two changes that have a considerable effect on their development and the pattern of the pharyngeal arch. First, each aorta grows cranially beyond the point at which the first artery of the pharyngeal arch joins it, while maintaining a close relation to the developing neural plate. Next, the dorsal aortas fuse, gradually displacing from lateral to medial, immediately caudal of the pharyngeal arches, in the direction of their caudal extremities to form a single medial vessel located between the developing intestine, ventrally, and the notochord, dorsally. The fusion extends as the embryo continues to grow. This fusion of the dorsal aortas starts anteriorly and propagates in the posterior direction.^3,6,7^

Along their path, each dorsal aorta emits small branches to the intersegmental plexuses between the somites, in addition to the vitelline branches to the corresponding side of the vitelline sac. Caudally, it also emits a large umbilical branch. The external and internal iliac arteries on each side emerge as branches of the intersegmental plexuses. In the sacral region, the intersegmental arteries are highly modified, but in the superior lumbar and thoracic regions, these arteries persist throughout life as the intercostal and lumbar arteries, arranged in series.^6^

Two patterns of persistent double dorsal aorta are described in the literature.^3,8^ Trubnikov et al.^9^ and Khristova et al.^10^ describe a descending aorta pattern in which there is a double lumen separated by a central dividing septum. The second, more prevalent pattern, has been described by Mosquera and Micarelli,^4^ Eibach and Walter,^11^ Formanek et al.,^12^ Brew et al.,^13^ Karadeli and Ulu,^8^ Chang and Rubin,^14^ Edwards et al.,^15^ Jie et al.,^16^ Mills and Gest^3^ and Hurtado et al.,^5^ and consists of complete separation of the two portions of the dorsal aorta.

The most observed variation within the category of complete separation of the dorsal aortas consists of presence of a principal dorsal aorta in conjunction with an accessory ascending aorta, which, in the majority of cases, runs to supply the posterior intercostal arteries.^3,11-16^ The anomaly presented in the current report is similar to the pattern reported by Mosquera and Micarelli,^4^ who described complete separation of two dorsal aortas into branches of the same caliber: one ventral and the other dorsal.

It is not rare for congenital anomalies of other sites to be found in conjunction with persistent dorsal aortas. These malformations primarily occur in the renal, vascular, or skeletal systems, particularly in the axial skeleton, in isolation or not. Additional aberrations described include cardiac, pulmonary, glandular, intestinal, and genital variations.^3,4,9,11-16^ In conjunction with the embryological defect seen in this case, the patient also had an anomalous origin of the inferior mesenteric artery, which formed from the anterior wall of the posterior branch of the dorsal aorta. Absence of other associated malformations is an exceptional condition, which has only been reported by Karadeli and Ulu^8^ and Khristova et al.^10^

The present study describes a unique pattern, with characteristics that had not been observed previously. In this case, the congenital variation was seen on an imaging exam (angiotomography), as in the majority of previous reports^5,8,11-15^ However, four cases, described by Mills and Gest,^3^ Trubnikov et al.,^9^ Mosquera and Micarelli,^4^ and Khristova et al.,^10^ were incidental findings in autopsies.

Studies using fish and birds as embryological models have proposed possible failures of mechanisms related to vasculogenic signaling pathways. These failures may occur because of inappropriate expression or suppression of positive or negative regulators associated with aortic fusion, causing persistence of two paired dorsal aortas.^3^
