# Supplementary material for: Case report: persistent double dorsal aorta
Source: J Vasc Bras. 2024 May 10;23:e20230150. doi: 10.1590/1677-5449.202301502 (PMC11129854; doi:10.1590/1677-5449.202301502)
Supplement: Translation

# Translation: Relato de caso: aorta dorsal dupla persistente

**DOI:** 10.1590/1677-5449.202301501

**Authors:** Paulo Henrique Alves Togni, Ernani Alves de Oliveira, Eduardo Milani Mora, Paulo Eduardo Borher Moreira, Bruno Previdelli Coghi, Guilherme Augusto Paro

**Affiliations:** 1 Centro Universitário Padre Albino – UNIFIPA, Catanduva, SP, Brasil.

**Keywords:** variação anatômica, aorta abdominal, tomografia computadorizada, anomalia congênita

## Abstract

A aorta dorsal dupla persistente é uma anomalia congênita extremamente rara, com apenas 13 casos publicados até o presente momento. Este estudo tem como objetivo apresentar a variação embriológica observada em aorta abdominal do paciente. A descrição anatômica foi realizada a partir da revisão de prontuário eletrônico e exames de imagem. Desta forma, o presente caso refere-se a um paciente idoso de 79 anos admitido em atendimento hospitalar por queixa de dor em membro inferior esquerdo ao repouso. Foi solicitada a internação do mesmo, assim como exames laboratoriais e de imagem. Obtida como um achado de imagem a partir de angiotomografia, a variação consiste na completa separação em duas porções: uma ventral e outra dorsal, de calibres diferentes, da aorta abdominal, ao nível da terceira vértebra lombar. Isso ocorre em conjunto com a origem anômala da artéria mesentérica inferior.

## INTRODUÇÃO

Aberrações congênitas e variantes do arco aórtico compreendem um amplo espectro de anomalias do desenvolvimento, sendo relativamente comuns e ocorrendo em 0,5 a 3% da população, associadas ou não a defeitos cardíacos^1,2^ . Apesar disso, defeitos embriológicos da aorta dorsal são excepcionais^3^ .

A persistência da aorta dorsal dupla é uma anomalia extremamente rara. O primeiro caso foi descrito em 1975 por Mosquera e Micarelli^4^ , e desde então apenas alguns casos foram relatados. Pela literatura, são descritas duas variantes: o padrão mais usualmente visto consiste na completa separação das duas porções da aorta dorsal. Já na outra variante, a aorta descendente apresenta um duplo lúmen devido à presença de um septo divisor central^3^ .

O presente trabalho foi aprovado pelo Comitê de Ética em Pesquisa do Centro Universitário Padre Albino (UNIFIPA), sob o número de Certificado de Apresentação de Apreciação Ética 68416423.0.0000.5430, e número do parecer consubstanciado 5.994.485.

## DESCRIÇÃO DE CASO

O paciente era do sexo masculino, branco, de 79 anos de idade. Foi admitido em serviço de urgência e emergência devido a dor em membro inferior esquerdo ao repouso, com piora há aproximadamente 10 dias. No exame físico, o paciente estava em bom estado geral, corado, hidratado e com perfusão periférica preservada. Havia presença de necrose no quinto dedo do pé esquerdo sem sinais inflamatórios. O paciente tinha antecedentes pessoais de hipertensão arterial sistêmica e doença arterial obstrutiva periférica, e era tabagista de longa data. Como conduta inicial, foram solicitados exames laboratoriais e de imagem, além de internação do mesmo. Durante a internação, foi administrada uma injeção de 60 mg de enoxaparina em uma seringa de 0,6 mL, além de analgesia com uma ampola de 100 mg de Tramadol e uma de Dipirona 1 g injetáveis, e membro inferior esquerdo foi mantido aquecido. Os exames laboratoriais indicaram sódio de 136 mmol/L, sem alterações nos demais parâmetros. Na ecografia com Doppler, as artérias à esquerda apresentaram fluxo monofásico na femoral comum, com oclusão da femoral em segmento proximal e médio. Poplítea com fluxo monofásico; tibial e fibular com fluxo monofásico, com oclusão da tibial posterior em segmento distal. Paciente permaneceu internado em leito de enfermaria para tratamento clínico por 13 dias sob cuidados gerais. Durante a internação, realizou-se fisioterapia nas disfunções vasculares periféricas e para transtorno respiratório sem complicações sistêmicas. No sétimo dia de internação, foram solicitadas aortografia e arteriografia de membro inferior esquerdo que evidenciou aorta abdominal exibindo morfologia e fluxo normais, com irregularidades parietais difusas.

No 13° dia, apesar do tratamento clínico, o paciente não apresentou melhora no membro inferior esquerdo, mantendo necrose, agora com discreta saída de secreção amarelada, sem hiperemia ou calor local, e pulsos não palpáveis. Para avaliação de transferência para serviço especializado de cidade vizinha, solicitou-se uma angiotomografia computadorizada no 13° dia. Pela angiotomografia, cortes tomográficos axiais do abdome foram realizados com a injeção do meio de contraste endovenoso, interessando apenas a fase arterial com opacificação da aorta abdominal e duplicação da porção inferior da mesma. A unidade anterior apresentava calibre de 2,6 cm e bifurcava-se em artérias ilíacas externas. Já a unidade posterior apresentava calibre de 2,2 cm, originando a artéria mesentérica inferior, que se encontrava parcialmente trombosada e bifurcava-se em artérias ilíacas internas (Figuras 1 e 2). As artérias ilíacas internas estavam parcialmente trombosadas, notadamente à direita, sendo que parte de seus ramos distais eram reperfundidos através de colaterais provenientes das artérias ilíacas externas ipsilaterais. As artérias intercostais posteriores originaram-se diretamente do segmento da aorta torácica. Não foram observadas anomalias de outros sítios pela tomografia. Decidida a transferência do paciente naquele dia, para serviço em cidade vizinha, solicitou-se jejum após as 18 horas. O paciente manteve acompanhamento em serviço de atendimento na cidade vizinha.

**Figura 1:** (a) Imagem de tomografia computadorizada (TC) em janela de abdome no plano axial; (b) Imagem de TC em janela de abdome no plano sagital mostrando duplicação inferior da aorta abdominal em unidade anterior (seta entalhada) e posterior (seta preta), a partir do nível de L3; (c) Imagem de TC em janela de abdome no plano axial mostrando duplicação inferior da aorta abdominal, com unidade anterior apresentando calibre de 2,6 cm, bifurcando-se em artérias ilíacas externas (cabeça de seta); e unidade posterior apresentando calibre de 2,2 cm, bifurcando-se em artérias ilíacas internas (trombosadas no estudo); (d) Imagem de TC em janela de abdome no plano axial mostrando duplicação da porção inferior da aorta abdominal, com unidade posterior originando a artéria mesentérica inferior (seta fina).

**Figura 2:** Imagens seriadas de TC em “volume-rendered” no plano frontal (a) e lateral esquerdo (b), mostrando duplicação da porção inferior da aorta abdominal que bifurca-se em artérias ilíacas externas (cabeça de seta) e unidade posterior bifurca-se em artérias ilíacas internas (trombosadas no estudo).

## DISCUSSÃO

A persistência da aorta dorsal dupla é uma condição extremamente rara, com apenas 12 casos descritos até o presente momento^3,5^ . A revisão bibliográfica realizada por Mills e Gest^3^ compilou 11 casos, datados de 1975 a 2016, e apenas um outro caso foi reportado em 2022, por Hurtado et al.^5^ .

Na embriologia da formação do sistema cardiovascular, os vasos embrionários primários são as aortas primitivas direita e esquerda, as quais se conectam cranialmente ao coração nascente por meio dos tratos de saída. Já as suas extremidades caudais alongam-se por meio da conexão com o plexo vascular no mesoderma esplâncnico. Essas duas aortas dorsais emparelhadas ficam abaixo do mesoderma da placa lateral e percorrem todo o comprimento do embrião. Logo após o seu aparecimento como vasos pareados separados, as aortas dorsais sofrem duas mudanças que têm um efeito considerável no seu desenvolvimento e no padrão do arco faríngeo. Em primeiro lugar, cada aorta cresce cranialmente além do ponto em que a primeira artéria do arco faríngeo se junta a ela, ademais, apresenta estreita relação com a placa neural em desenvolvimento. Em segundo lugar, as aortas dorsais se fundem, translocando-se gradualmente de lateral para medial, imediatamente caudal aos arcos faríngeos, em direção às suas extremidades caudais para formar um único vaso mediano situado entre o intestino em desenvolvimento, ventralmente, e a notocorda, dorsalmente. À medida que o crescimento do embrião prossegue, essa fusão se estende. Este evento de fusão entre as aortas dorsais começa anteriormente e se propaga na direção posterior^3,6,7^ .

No decorrer de seu trajeto, cada aorta dorsal emite pequenos ramos para os plexos intersegmentares entre os somitos, além de ramos vitelinos para o lado correspondente do saco vitelino. Caudalmente, emite também um grande ramo umbilical. As artérias ilíacas externa e interna, de cada lado, surgem como ramos dos plexos intersegmentares. Na região sacral, as artérias intersegmentares são muito modificadas, entretanto, nas regiões torácica e lombar superior, essas artérias persistem ao longo da vida como as artérias intercostais e lombares dispostas em série^6^ .

São descritos na literatura dois padrões de persistência da aorta dorsal duplicada^3,8^ . Trubnikov et al.^9^ e Khristova et al.^10^ reportaram um padrão de aorta descendente de duplo lúmen com a presença de um septo divisor central. O segundo, mais prevalente, descrito por Mosquera e Micarelli^4^ , Eibach e Walter^11^ , Formanek et al.^12^ , Brew et al.^13^ , Karadeli e Ulu^8^ , Chang e Rubin^14^ , Edwards et al.^15,^ Jie et al.^16^ , Mills e Gest^3^ e Hurtado et al.^5^ , consiste na completa separação das duas porções da aorta dorsal.

A variação mais observada dentre a categoria da completa separação das aortas dorsais compõe-se da presença de uma aorta dorsal principal em associação a uma aorta acessória ascendente, a qual, na maioria dos casos, dirige-se para irrigar as artérias intercostais posteriores^3,11-16^ . A anomalia apresentada nesse relato assemelha-se ao padrão reportado por Mosquera e Micarelli^4^ , onde descreveram a completa separação das duas aortas dorsais em ramos de mesmo calibre: um ventral e outro dorsal.

Não raramente, anomalias congênitas de outros sítios associam-se ao padrão de persistência das aortas dorsais. As malformações ocorrem, principalmente, no sistema renal, vascular ou esquelético, em especial no esqueleto axial, associadas ou não. Além disso, são descritas aberrações cardíacas, pulmonares, glandulares, intestinais e genitais^3,4,9,11-16^ . Em associação ao defeito embriológico apresentado por esse caso, observou-se uma anomalia na origem da mesentérica inferior, que formou-se da parede anterior do ramo posterior da aorta dorsal. A ausência de outras malformações associadas é uma condição excepcional, reportada apenas por Karadeli e Ulu^8^ e Khristova et al.^10^ .

O presente estudo descreve um padrão único, com características até então não observadas. Nesse caso, a variação congênita foi visualizada a partir de exame de imagem (angiotomografia), assim como a maioria dos relatos anteriores^5,8,11-15.^ Contudo, quatro casos, descritos por Mills e Gest^3^ , Trubnikov et al.^9^ , Mosquera e Micarelli^4^ , Khristova et al.^10^ , foram achados acidentais de autópsia.

A partir de estudos em peixes e aves como modelos embriológicos, foram propostas possíveis falhas em mecanismos relacionados às vias de sinalização vasculogênica. Estas falhas podem ocorrer devido à expressão ou supressão inapropriada de reguladores positivos ou negativos associados à fusão aórtica, determinantes na persistência das duas aortas dorsais pareadas^3^ .
